# Assessment of the Neutrophil-Lymphocyte Ratio in Classic Hodgkin Lymphoma Patients

**DOI:** 10.12669/pjms.35.5.601

**Published:** 2019

**Authors:** Ali Dogan, Sinan Demircioglu

**Affiliations:** 1 Ali Dogan, Department of Hematology, Van Yuzuncu Yil University, Faculty of Medicine, Van, Turkey; 2 Sinan Demircioglu, Department of Hematology, Van Yuzuncu Yil University, Faculty of Medicine, Van, Turkey

**Keywords:** Hodgkin lymphoma, Lymphocyte, Neutrophil, Neutrophil-lymphocyte ratio, Prognosis, Stage

## Abstract

**Background and Objective:**

Besides known risk scoring systems, studies have recently been conducted in relation to NLR to estimate the prognosis of HL. Some studies found a relationship of NLR with PFS and OS. Our objective was to investigate whether NLR, as an inexpensive and easily accessible test, is a prognostic marker for cHL, as in several previous studies.

**Methods:**

The study included 232 patients in the age range of 18 to 88 years who were diagnosed with classic Hodgkin Lymphoma and received ABVD chemotherapy and/or radiotherapy at Van Yuzuncu Yil University Hematology Clinic from 2000-2018. Analyses were conducted on the disease stage, risk scores, treatment responses and relapse statuses at the time of diagnosis based on the patients’ NLR values at the time of diagnosis.

**Results:**

The mean age of the patients was 39.27 ± 15.90, while 38.8% were female and 61.2% were male. The NLR value at the time of diagnosis was significantly related to stage (p:0.013), early-stage risk score (p:0.022) and treatment response (p:0.032). The cutoff value of NLR was found as 4.23. The Hb value at the time of diagnosis was significantly related to stage (p:0.00), early-stage risk score (p:0.007), treatment response (p:0.006) and the latest status of patients (p:0.005).

**Conclusion:**

High NLR values were found to be significantly related to disease stage, early-stage risk scoring and response to the treatment. These findings need to be supported by prospective studies with larger samples for these data to be used prognostic scores.

## INTRODUCTION

Hodgkin lymphoma (HL) is a rare malignity that involves lymph nodes and the lymphatic system. Most patients are diagnosed between the ages of 15 and 30, and a second peak is observed in adults of 55 years of age or older.^1^ The World Health Organization (WHO) classifies HL into two main groups as classic Hodgkin lymphoma (cHL) and nodular lymphocyte-predominant Hodgkin lymphoma (NLPHL).^2^ Among all HL cases in Western countries, 95% correspond to cHL, while 5% correspond to NLPHL. cHL has 4 sub-types as nodular sclerosis classic HL (NSHL), mixed cellularity classic HL (MCHL), lymphocyte rich classic HL (LRHL) and lymphocyte depleted classic HL (LDHL).

The prognostic factors of early-stage HL were defined as the presence of a large mediastinal mass, high sedimentation rate, involvement of multiple modal regions, extra-nodal involvement, age of older than 50 years or presence of a massive splenic disease.^3^,^4^ For advanced-stage HL, an international prognostic scoring system which consists of seven variables (age >45, presence of stage-4 disease, male sex, leukocytosis >15 000/mm^3^, lymphopenia <600/mm^3^, albumin <4.0 g/dL, hemoglobin (Hb) <10.5 g/dL) was defined.^5^ These diagnostic scoring systems are used in both predicting the course of the disease and planning treatment.

The prognostic significance of the neutrophil/lymphocyte ratio (NLR) is known in recent times in relation to some solid organ malignity cases. It was shown that NLR is a prognostic factor in diffuse large B-cell lymphoma (DLBCL).^6^-^8^ In addition to this, a few studies have examined the potential prognostic value of NLR in cHL.^9^-^11^ The aim of this study was to investigate whether NLR, as an inexpensive and easily accessible test, is a prognostic marker for cHL, as in several previous studies.

## METHODS

### Study design

The study included 232 patients in the age range of 18 to 88 years who were diagnosed with classic Hodgkin Lymphoma and received ABVD chemotherapy and/or radiotherapy. The patients’ demographic data, stages at the time of diagnosis based on the Ann Arbor classification, risk groups, laboratory values, treatment responses, relapse statuses and latest statuses were determined retrospectively. Stages-1 and 2 were defined as early stage, while stages-3 and 4 were defined as advanced stage. The prognostic classification of early stages was made by the risk classification of the European Organization for the Research and Treatment of Cancer (EORTC).^12^ Negative risk factor was defined as the presence of at least one of these criteria: age (>50), sedimentation (>50 mm/s), mediastinal thorax rate (MTR) (>0.35) and/or number of nodal regions (>4). The advanced stage classification was made by the international prognostics scoring (IPS) method.^5^ Negative risk factor was defined as at least three of these criteria: albumin (<4 g/dl), Hb (<10.5 g/dl), male sex, age (>45), stage-4 disease, leukocytosis (>15.000/mm^3^) and/or lymphopenia (<600/mm^3^).

### Statistical analysis

While our study expresses the continuous variables with their descriptive characteristics as mean, standard deviation, minimum and maximum values, the categorical variables are expressed in terms of frequencies and percentages. NLR was found by dividing the number of neutrophils by the number of lymphocytes. While analyzing the data, Kolmogorov-Smirnov normality test was used to determine the suitability of the continuous variables for normal distribution. The data that were normally distributed were analyzed by using independent-samples t-test, while the non-normally distributed data were analyzed by using Mann-Whitney U test. The threshold values of NLR and Hb were determined by ROC curve analysis. A p-value of <0.05 was accepted to be statistically significant. The analyses were carried out using SPSS 24.

### Ethics committee approval

The study was approved by the Van Yuzuncu Yil University Local Ethics Committee (protocol number: 09.11.2018/08).

## RESULTS

**Fig.1:**
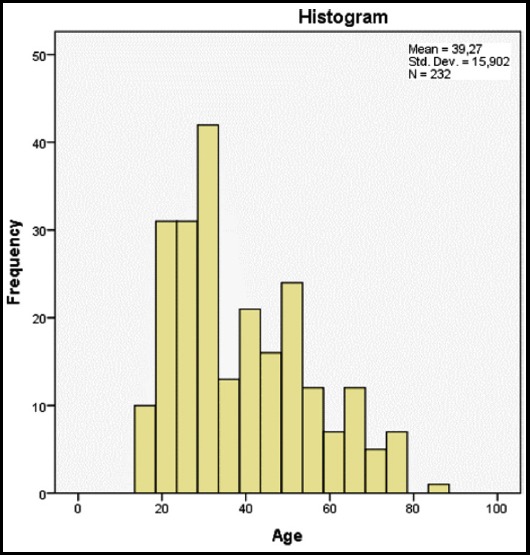
Distribution by age at diagnosis.

The study included 232 patients who were diagnosed with classic Hodgkin Lymphoma. The mean age of the patients was found as 39.27 ± 15.90. The age histogram is shown in Fig.1. The disease stages, prognostic characteristics, treatment responses, relapse and latest statuses are shown in Table-I while the laboratory findings are shown in Table-II.

**Table I:** Participant features.

		n	%
Sex	Female	90	38.8
	Male	142	61.2
HL subtypes	Nodular sclerosis	129	55.6
	Mixed cellularity	52	22.4
	Lymphocyte rich	37	15.9
	Lymphocyte depleted	14	6.0
Stage	Stage 1	38	16.4
	Stage 2	62	26.7
	Stage 3	88	37.9
	Stage 4	44	19.0
	Early stage (stage I to II)	101	43.5
	Advanced stage (stage III to IV)	131	56.5
Early stage	Favorable risk	46	45.5
	Unfavorable risk	55	54.5
Advanced stage	Favorable risk	8	6.1
	Unfavorable risk	123	93.9
Treatment response	CR	200	86.2
	PR	4	1.7
	SD/PD/EF	28	12.1
Recurrence status	No recurrence	191	82.3
	Recurrence	41	17.7
Final status	Lives	215	92.7
	Death	17	7.3

CR: Complete response, PR: Partial response, SD: Stable disease, PD: Progressive disease, EF: Early failure.

**Table II:** Serum levels of the numerical variables analyzed.

	Mean	Min.	Max.	Std. Deviation(SD)
Leucocyte count	8952	800	25700	4821.53
Neutrophil count	6586	100	16000	6354.63
Lymphocyte count	1860	100	14000	1408.05
NLR	5.05	0.04	42	5.58
Hemoglobin	12.47	5.4	18.90	2.42
Thrombocyte count	150000	86900	302836	144715.21

The NLR value at the time of diagnosis was significantly related to stage (p:0.013), early-stage risk score (p:0.022) and treatment response (p:0.032). It had no significant relationship with disease prognostic characteristics (p:0.38), relapse status (p:0.51) or latest status (p:0.87) regarding the advanced stages. The mean NLR value was found as 3.94 ± 3.70 in the early stages and 5.91 ± 6.56 in the advanced stages. While the mean NLR value was found as 2.77 ± 1.61 in the group with early-stage positive prognostic characteristics, it was found as 4.58 ± 0.51 in the early-stage negative risk group. This value for the group that responded to the treatment (CR+PR) was 4.97 ± 5.76, while it was 5.63 ± 4.09 in the group that did not respond (Table-III).

**Table III:** NLR with stage, prognostic features, treatment response and relapse status of the relationship

		NLR			

		Mean	Std. Deviation	Z value	P value
Stage	Early Stage	3.94	3.70	-0.483	0.013
	Advanced stage	5.91	6.56		
Early stage	Favorable risk	2.77	1.61	-2.294	0.022
	Unfavorable risk	4.58	3.72		
Advanced stage	Favorable risk	5.12	2.29	-0.865	0.387
	Unfavorable risk	5.96	6.75		
Treatment response	CR+PR	4.97	5.57	-2.142	0.032
	SD/PD/EF	5.63	4.09		
Recurrence status	No recurrence	4.90	5.35	-0.645	0.519
	Recurrence	5.76	6.55		
Final status	Lives	5.07	5.77	-1.710	0.87
	Death	4.84	2.16		

**Fig.2:**
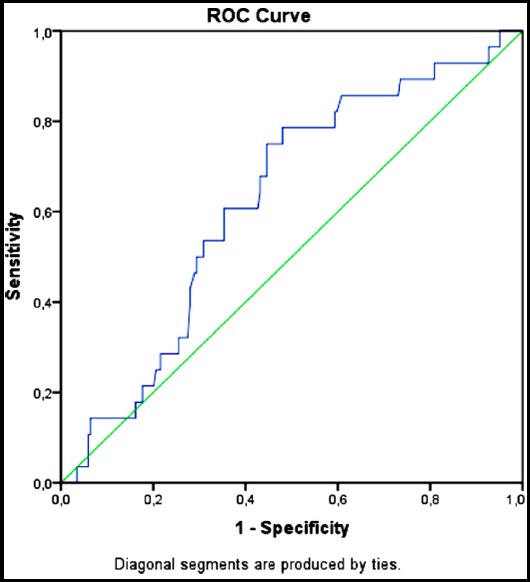
NLR, ROC curve

The ROC analysis demonstrated an area under the curve (AUC) of 0.625 (Fig.2). A cut-off value of 4.23 for NLR was able to predict treatment response rate with a sensitivity and specificity of 60% and 65%, respectively (likelihood ratio-LR=1.72).

**Fig.3:**
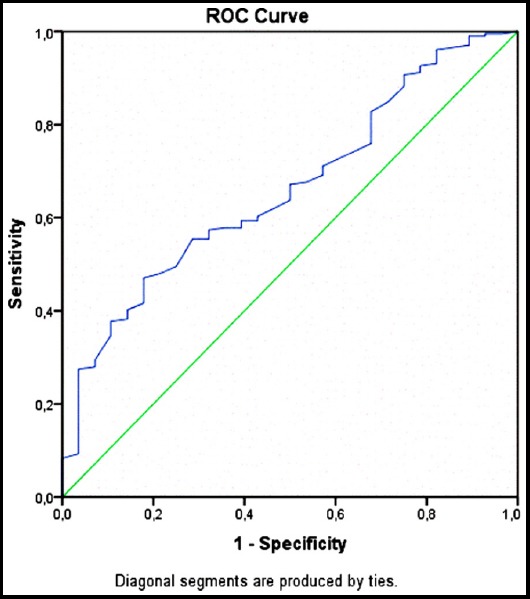
Hb, ROC curve

The ROC analysis demonstrated an area under the curve (AUC) of 0.662 (Fig.3). A cut-off value of 12.25 g/dl for Hb was able to predict treatment response rate with a sensitivity and specificity of 59% and 61%, respectively (likelihood ratio-LR=1.50).

The Hb value at the time of diagnosis was also significantly related to stage (p:0.00), early-stage risk score (p:0.007), treatment response (p:0.006) and the latest status of patients (p:0.005). The mean Hb level was found as 13.27 ± 2.20 g/dl in the early stages (1 and 2) and 11.86 ± 2.42 g/dl in the advanced stages. The mean Hb value was 13.93 ± 1.96 g/dl in the group that had early-stage positive prognostic characteristics, while it was found at 12.69 ± 2.29 g/dl in the group of negative risk. This value for the group that responded to the treatment (CR+PR) was Hb 12.65 ± 2.36 g/dl, while the group that did not respond (SD/PD/EF) had a mean value of 11.18 ± 2.54 g/dl. The mean Hb value was found as 12.61 ± 2.38 g/dl in the patients who survived and 10.78 ± 2.37 g/dl in the patients who died (Table-IV).

**Table IV:** Hb with stage, prognostic features, treatment response and relapse status of the relationship.

		Hb			

		Mean	Std.Deviation	Z value	P value
Stage	Early Stage	13.27	2.20	-4.614	0.000
	Advanced stage	11.86	2.42		
Early stage	Favorable risk	13.93	1.96	-2.688	0.007
	Unfavorable risk	12.69	2.29		
Advanced stage	Favorable risk	12.78	1.05	-1.188	0.235
	Unfavorable risk	11.80	2.47		
Treatment response	CR+PR	12.65	2.36	-2.273	0.006
	SD/PD/EF	11.18	2.54		
Recurrence status	No recurrence	12.54	2.48	-1.104	0.269
	Recurrence	12.14	2.15		
Final status	Lives	12.61	2.38	-2.781	0.005
	Death	10.78	2.37		

## DISCUSSION

Neutrophil/lymphocyte ratio is accepted as a simple and strong parameter related to prognosis in cancer patients for assessing both inflammatory (neutrophil) and immune (lymphocyte) responses. In this context, several studies proved the prognostic significance of NLR, which was check on diagnosis and before treatment, in patients with solid organ tumors.^13^ It was also shown in hematological malignity cases such as diffuse large B-cell lymphoma that high NLR is related to poor prognosis.^7^,^8^,^14^,^15^ The reason for the correlation of NLR with prognosis is largely unknown. In addition to this, it was reported that normal neutrophils may suppress the function of T-cells, and activated neutrophils increase the level of arginase 1, which leads to suppression of T-cells.^16^ In addition to contributing to the immune suppression of T-cells, neutrophils may also show characteristics that support tumors such as inducing angiogenesis and increasing tumor metastasis by raising the expression of matrix metalloproteinase.^9^

We also investigated the relationships NLR in cHL at the time of diagnosis had with disease stages, risk scores, treatment responses and statuses of relapse. In our study, the NLR values at the time of diagnosis were found to be higher in the advanced-stage patients, early-stage negative risk group and patients who did not respond to treatment. There was no significant relationship between high NLR values and relapse status.

A study by Marcheselli et al. on 990 nodular sclerosis-cHL patients reported that the value of NLR >6 was associated with both poor progression-free survival (PFS) and overall survival (OS) in both early-stage and advanced-stage cases. When they compared those with NLR >6 and those with NLR ≤6 at the time of diagnosis, they respectively found the 5-year rates of PFS as 75% and 84% and OS as 88% and 92%.^10^ A study by Koh et al. that was conducted with 312 cHL patients showed that NLR values of higher than 4.3 were related to poor OS rates in advanced-stage patients.^9^ Another study on early-stage cHL found 2-year freedom from progression (FFP) as 82.2% in those with NLR ≥6.4 and 95.7% in those with NLR <6.4, associating high NLR levels with poor FFP.^11^ It was not possible in our study to conduct PFS and OS analyses as the monitoring time of the patients was not sufficient. On the other hand, we found the NLR cutoff value in terms of responding to treatment as 4.23.

Romano et al.’s study on 180 cHL patients found NLR higher in comparison to the control group (5.0 vs. 1.6). They also found a relationship between increased NLR and advanced stage, increased number of neutrophils, reduced number of lymphocytes and increased value of erythrocyte sedimentation. They showed NLR was higher in those with positive PET-CT results in comparison to those with negative results after two cures of ABVD chemotherapy (7.4 vs. 4.8). NLR was found to be lower in patients who showed complete response to the treatment in comparison to those who did not (4.8 vs. 7.7). When those with NLR >6 and those with NLR ≤6 were compared, the researchers found 5-year PFS was 70.1% and 86.6%, respectively.^17^ Likewise, we also found a relationship between high NLR and advanced-stage disease.

Advanced-stage cHL’s disease risk assessment is made by IPS. IPS has seven components. 5-year progression-free survival is 84% if there is no risk factor, while it is 42% if there are 5-7 risk factors. One of the components of IPS is Hb<10.5 g/dl.^5^ In our study, Hb was significantly lower in the advanced-stage disease, early-stage poor risk and non-responsive groups. However, although the Hb value in the advanced-stage negative risk group was lower, there was no statistical significance in the difference. Our study found the Hb cutoff value as 12.25 g/dl.

### Limitations of the study

It was a study with a retrospective design, the number of cases was small, and it was not possible to conduct PFS and OS analyses due to the insufficient duration of monitoring.

## CONCLUSION

NLR is an inexpensive and easily accessible test whose prognostic significance has been shown in several studies. Although it was not possible in our study to investigate the relationship of NLR with PFS and OS due to the short times of monitoring the patients, we showed that it is related to stage, early-stage risk status and treatment response.
